# Counterfactual Anonymous Quantum Teleportation in the Presence of Adversarial Attacks and Channel Noise

**DOI:** 10.3390/s22197587

**Published:** 2022-10-06

**Authors:** Saw Nang Paing, Jason William Setiawan, Shehbaz Tariq, Muhammad Talha Rahim, Kyesan Lee, Hyundong Shin

**Affiliations:** Department of Electronics and Information Convergence Engineering, Kyung Hee University, Yongin 17104, Korea

**Keywords:** counterfactual, robustness, security, correctness, anonymity, noise-tolerance level

## Abstract

Hiding the identity of involved participants in the network, known as anonymity, is a crucial issue in some cryptographic applications such as electronic voting systems, auctions, digital signatures, and Byzantine agreements. This paper proposes a new anonymous quantum teleportation protocol based on counterfactual communication where no information-carrying particles pass through the channel. It is achieved by the distribution of a counterfactual entanglement among the participants in the network followed by the establishment of an anonymous entanglement between the sender and the receiver. Afterwards, the sender can anonymously teleport a quantum state to the receiver by utilizing the anonymous entanglement. However, the practicality of the anonymous quantum network mainly calls for two performance measures—robustness against adversarial attacks and noisy environments. Motivated by these demands, firstly, we prove the security of our proposed protocol and show that it achieves both the sender and receiver’s anonymity in the presence of active adversaries and untrusted parties. Along with anonymity, we also ensure the correctness of the protocol and the privacy of the teleported qubit. Finally, we analyze the robustness of our proposed protocol under the presence of channel noise and compare its fidelity with those of the conventional protocols. The main advantage of our proposed protocol is that it can provide useful anonymous quantum resources for teleportation under noisy environment with a higher security compared to previous protocols.

## 1. Introduction

Quantum cryptography has brought a lot of interesting, secure communication protocols such as quantum key distribution [1], quantum secure direct communication [2], quantum secret sharing [3], quantum private comparison [4], etc., under the laws of quantum mechanics. These protocols ensure the unconditional security of the transmitted message, i.e., the content of the transmitted information is learned only by the sender and the receiver. At the same time, the irrelevant participants or adversaries get no knowledge of it. However, not all communication applications are confined only to the security of the message. Some cryptographic applications, such as electronic voting, auction, digital signature, Byzantine agreement, etc., require hiding the participants’ identities to complete the task without bias. This hiding of participants’ identities while accomplishing the communication task is known as anonymity. Hence, anonymity is important in securing the identity of communicating parties, just as absolute security is crucial to the confidentiality of the secret message.

The first classical anonymous protocol proposed in [5] demonstrated the unconditional tracelessness of the message sender and receiver. This protocol determines if a dinner bill is paid anonymously without disclosing any other information. Each cryptographer at the dining table secretly flips an unbiased coin with their right neighbor; hence, they can see the coin they flipped and the coin their left neighbor flipped. The cryptographers then announce the state of the two coins, the same or different sides. If one of the cryptographers is the payer, they reveal the opposite result. After that, they compute the sum of all the announcements. If the sum equals one, one of them pays the dinner bill. Hence, this protocol enables the anonymous transmission of one bit that confirms the payment.

Later, by incorporating quantum mechanics, it was expanded to the quantum version of anonymous transmission [6]. Although this protocol used quantum resources, we could only use it for the transmission of classical messages anonymously. To address this limitation, a Greenberger–Horne–Zeilinger (GHZ) based anonymous communication protocol that allowed the transmission of quantum information was proposed in [7]. The key concept behind this idea was to distribute an anonymous entanglement between the sender and the receiver, followed by the desired communication task. In addition to the multipartite entanglement-based protocols, an entanglement relay-based quantum anonymous transmission protocol was proposed [8]. The protocol was proved privacy-preserving and could enable long-distance anonymous quantum communication. However, most of the anonymous quantum communication protocols did not take channel noise into consideration. Hence, a W-state-based quantum anonymous transmission protocol that outperformed the GHZ state-based and entanglement relay-based protocols in the presence of noise was proposed [9]. To date, various anonymous quantum communication protocols have been proposed based on single particles, Bell states, GHZ states, and W states [10,11,12,13,14,15,16,17,18,19,20,21]. In order to employ these protocols in practical network scenarios, they must be able to provide the desired communication task under the presence of adversaries and a noisy environment. However, all of these protocols have one thing in common—the particle that travels across the quantum channel to fulfill the communication task can be intercepted by adversaries, failing communication. In addition, most of these protocols do not provide a noise analysis.

To compensate for the aforementioned issues, we propose a new anonymous quantum teleportation protocol which makes use of counterfactual communication. By enabling the transmission of information without any information-carrying particle passing through the channel, counterfactual communication prevents attacks that rely on the intercepted information-carrying particle in the channel. Counterfactual communication arises from the interaction-free measurement, which infers the presence of a bomb without touching it with 25% probability [22]. With the integration of the quantum Zeno effect [23], this probability approaches unity and leads to the counterfactual transmission of the one-bit value. The gate that enables this kind of operation is called the quantum Zeno (QZ) gate. By inserting a QZ gate within another QZ gate, known as a chained quantum Zeno (CQZ) gate, the counterfactual communication of two-bit values is achieved [24]. Recently, counterfactual communication has been applied to different areas, including quantum cryptography, quantum computing, and quantum communication [25,26,27,28,29,30,31].

Most of the previous anonymous quantum communication protocols rely on the preshared entanglement among the participants in the network. In this work, we counterfactually distribute the entanglement among the participants and accomplish the task of anonymous quantum teleportation. In order to meet the requirements of practical quantum networks, we perform a security and noise analysis for our proposed protocol. The security of our proposed protocol is ensured by examining it under counterfactual man-in-the-middle attacks and Trojan horse attacks. For the noise analysis, we evaluate the performance of our proposed protocol in the presence of channel noise and compare it with the performance of relay-based and GHZ-based anonymous quantum communication. The rest of the paper is as follows: Section 2 describes the preliminaries followed by our proposed method. Section 3 analyzes the performance of the protocol with emphasis on its security, accuracy, privacy, and noise. Section 4 describes the application of our proposed protocol in an IoT network. Finally, Section 5 gives our conclusion.

## 2. Materials and Methods

### 2.1. Preliminaries

This section introduces the basic theorems and gates utilized in our counterfactual anonymous quantum teleportation protocol.

#### 2.1.1. Collision Detection

**Theorem** **1.** *The collision detection protocol allows the detection of the existence of multiple senders in one round of the protocol. The protocol starts by allowing each participant to input one bit. Let v represent the number of “1’s" inputted by all participants in the network. The protocol has three possible outcomes depending on the value of v: (i) no participant wants to perform the communication task (v=0), (ii) only one participant wants to perform the communication task (v=1), and (iii) more than one participant wants to perform the communication task (v≥2). If all the participants in the network are honest, the protocol outputs the correct result with a probability exponentially close to 1 [13]. The correctness of the protocol does not allow any individual participant to terminate the protocol. The adversary gains no additional information even if the protocol is implemented correctly, except for them allocating random bit values rather than “0" to all conspiring participants [32]. Due to its similarity with the veto protocol [33], the presence of a single corrupt participant will lead to the outcome corresponding to (iii), regardless of the inputs of the other participants. Hence, no cheating is possible, and the protocol succeeds in detecting collisions in the presence of adversaries.*

#### 2.1.2. Notification Protocol

**Theorem** **2.** *The notification protocol allows any participant in the network to notify other participants of their preference. Each participant outputs a private bit that indicates whether or not they have been notified at least once. The value of the bit is correctly calculated with a probability exponentially close to 1 [14,32,33].*

#### 2.1.3. Anonymous Broadcast of Classical Message

**Theorem** **3.** *When a sender anonymously broadcasts their message s to n participants in the network, it must meet the following criteria [32,33]:*

*1*  *Every participant in the network receives the message s;*
*2*  *The identity of the sender remains hidden from any adversary, i.e., if the adversary has control over t participants, the probability that they can correctly guess the identity of the sender is no more than $1/\left(n-t\right)$;*
*3*  *Any malicious behavior against the protocol is discovered.*

#### 2.1.4. CQZ Gate

**Figure 1:**
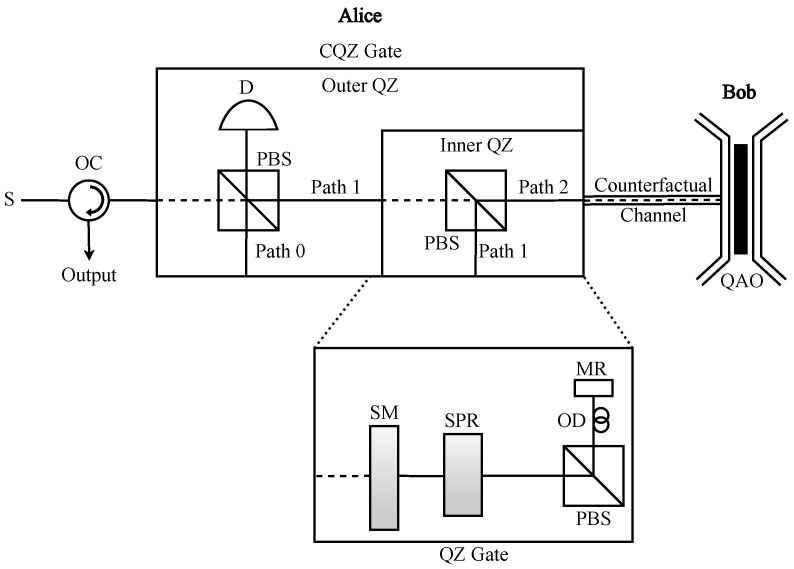
Chained quantum Zeno (CQZ) gate. It is a nested version of the quantum Zeno (QZ) gate where Alice inputs a source photon (S), which can be an H- or V-polarized photon. It is made up of a set of optical devices, including switchable mirrors (MR), switchable polarization rotators (SPR), polarizing beam splitters (PBS), optical delays (OD), mirrors (MR), optical circulator (OC), and detectors (D). Once the photon enters into the CQZ gate, it gets collapsed into three different paths, namely path 0, path 1, and path 2, due to the SPRs and PBSs in the outer and inner QZ gates. Only the photon component in path 2 enters into the quantum channel. Bob, who is on the other side of the quantum channel, holds a quantum absorptive object (QAO), which is a superposition of absence and presence states of absorptive object (AO). He decides whether to absorb or not that photon component by inserting or not inserting AO. For a complete CQZ gate operation, the photon experiences *M* cycles of the outer QZ gate where each outer cycle consists of *N* cycles of the inner QZ gate. The logical operation of the CQZ gate is that it retains (rotates) the polarization of the incoming source photon in the absence (presence) of AO.

A chained quantum Zeno (CQZ) gate [24] is used to realize the logic of counterfactual communication, which is a nested version of the quantum Zeno (QZ) gate. We can implement it using optical components such as polarizing beam splitters (PBS), switchable polarization rotator (SPR), switchable mirrors (SM), mirrors (MR), optical delays (OD), and photon detectors (D), as shown in Figure 1. The gate that takes an H(V)-polarized photon as input is called H(V)-CQZ gate, respectively. To initiate the CQZ gate, Alice inputs the photon into the gate and it gets rotated by the SPR. Here, we denote SPR${}^{H(V)}$ as the SPR used in the H(V)-QZ gate, and its function can be described as

(1) $$\begin{array}{cc} |H(V)\rangle & \to \cos\theta |H(V)\rangle +\sin\theta |V(H)\rangle , \end{array}$$

(2) $$\begin{array}{cc} |V(H)\rangle & \to \cos\theta |V(H)\rangle -\sin\theta |H(V)\rangle , \end{array}$$

respectively, where θ is the rotation angle. Let us denote $\theta_{N}=\pi /2N$ and $\theta_{M}=\pi /2M$ as the angles rotated by the SPR in the inner and outer QZ gates, respectively, where N(M) represents the number of inner (outer) cycles. After the photon has been rotated by the SPR, the PBS separates it into two components; one component goes into path 0, where it gets stored for a certain period, and the other goes into the inner QZ gate via path 1. The photon component in path 1 gets rotated by another SPR and is again separated into two parts by another PBS; one component gets stored in path 1 while the other component is transmitted into the quantum channel through path 2.

Bob, on the other side of the transmission channel, decides whether to absorb or reflect the photon. If he decides to absorb the incoming photon, he inserts the absorptive object (AO) at their side of the transmission channel. Otherwise, he does nothing. If the photon component that enters the transmission channel is not absorbed by AO and reflected from Bob’s side, it combines with the photon component in path 1 and goes to the start of the inner QZ gate. Otherwise, the next inner cycle starts with only the photon component in path 1. After this procedure is repeated *N* times in the inner QZ gate, the photon component that comes out of the inner QZ gate combines with the photon component in path 0 at the PBS of the outer QZ gate. The resulting photon after the PBS is used again for the next cycle of the outer QZ gate. In case of the absence of AO, it is discarded at detector D to ensure counterfactuality. Finally, we can describe the state of the photon after the CQZ gate with *M* outer and *N* inner cycles in the absence and presence of AO as follows:

(3) $$\begin{array}{cc} Absence: & |H(V)\rangle \to \cos m\theta_{M}|H(V)\rangle +\sin m\theta_{M}|V(H)\rangle \overset{m=M}{\to }|H(V)\rangle , \end{array}$$

(4) $$\begin{array}{cc} Presence: & |H(V)\rangle \to \cos^{m-1}\theta_{M}\left(\cos\theta_{M}|H(V)\rangle +\sin\theta_{M}|V(H)\rangle \right)\overset{m=M}{\to }|V(H)\rangle . \end{array}$$

Thus, if Bob does not insert an AO, the polarization of the photon sent by Alice remains the same with probability $\eta_{1}=\cos^{2M}\theta_{M}$; otherwise, the photon with opposite polarization is resulted with probability $\eta_{2}=\prod_{m=1}^{M}{\left(1-\sin^{2}\left(m\theta_{M}\right)\sin^{2}\theta_{N}\right)}^{KN}$ [31]. These probabilities tend to 1 as *N* and *M* approach infinity.

### 2.2. Counterfactual GHZ State Distribution

**Figure 2:**
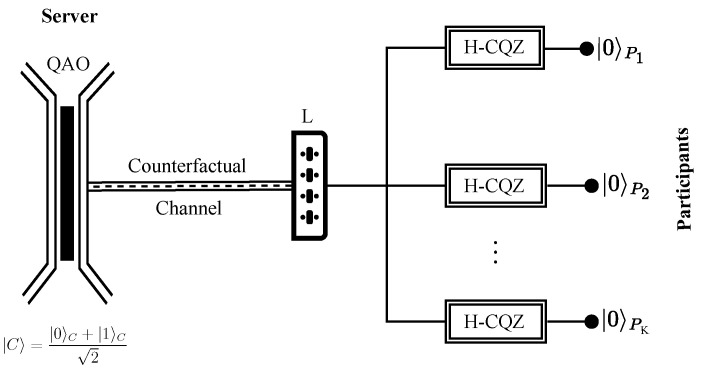
Counterfactual GHZ state distribution. Consider a network that consists of a server and *K* participants. The server holds a QAO which is in the state $|C\rangle =\frac{{|0\rangle }_{C}+{|1\rangle }_{C}}{2}$ while the *K* participants hold H-polarized photons denoted as ${|0\rangle }_{P_{i}}$ where $i\in \left\{1,2,...,K\right\}$. Each participant performs an H-CQZ operation upon establishing a connection with the server through the switch L. Once all the *K* participants have completed their respective H-CQZ operations in a consecutive manner, a K+1 partite GHZ state is distributed among the *K* participants and the server, as described in Equation (8).

Consider a network that consists of a server and *K* participants. The server prepares a quantum AO (QAO), which is the superposition of the absence and presence states of an AO, as $|C\rangle =\frac{{|0\rangle }_{C}+{|1\rangle }_{C}}{2}$. Meanwhile, each participant holds an H-polarized photon and prepares an H-CQZ gate. Here, ${|0\rangle }_{C}\left({|1\rangle }_{C}\right)$ represents the absence (presence) of AO and the H(V)-polarized photon is denoted as ${|0\rangle }_{P_{i}}\left({|1\rangle }_{P_{i}}\right)$ where $i\in \left\{1,2,...,K\right\}$. Using the tripartite counterfactual entanglement distribution in [34], we extend it to a multipartite case. As the server is responsible for the counterfactual GHZ state distribution, the H-CQZ gate of each participant $P_{i}$ is connected to the server via the switch *L* as shown in Figure 2.

To start the protocol, the server connects $P_{1}$ through the switch *L* and $P_{1}$ inputs their H-polarized photon ${|0\rangle }_{P_{1}}$ into the H-CQZ gate. As described in Section 2.1, the logical operation of the H-CQZ gate is that it completely rotates the polarization of the incoming photon in the presence of an AO and retains the polarization in the absence of a photon. If the photon is not lost during the operation of the CQZ gate, the initial combined state of the server and the participants becomes

(5) $$\begin{array}{c} |\psi_{0}\rangle =\left(\frac{{|0\rangle }_{C}+{|1\rangle }_{C}}{\sqrt{2}}\right)⊗{|0\rangle }_{P_{1}}⊗{|0\rangle }_{P_{2}}⊗\cdots ⊗{|0\rangle }_{P_{K}} \end{array}$$

and changes into

(6) $$\begin{array}{c} |\psi_{1}\rangle =\frac{1}{\sqrt{2}}\left(\sqrt{\eta_{1}}{|00\rangle }_{CP_{1}}+\sqrt{\eta_{2}}{|11\rangle }_{CP_{1}}\right)⊗{|0\rangle }_{P_{2}}⊗\cdots ⊗{|0\rangle }_{P_{K}}, \end{array}$$

where $\eta_{1}$ and $\eta_{2}$ correspond to the success probabilities of counterfactual communication in the presence and absence of an AO, respectively. The CQZ operation between the server and $P_{1}$ establishes entanglement between them while the qubits of the other participants remain separated.

Next, the server closes its connection with $P_{1}$ and establishes a new connection with $P_{2}$. $P_{2}$ sends their photon towards their respective H-CQZ gate, which undergoes *M* outer and *N* inner cycles of the CQZ gate. If it does not get discarded after the gate, the state $|\psi_{1}\rangle$ becomes

(7) $$\begin{array}{c} |\psi_{2}\rangle =\frac{1}{\sqrt{2}}\left(\sqrt{\eta_{1}^{2}}{|000\rangle }_{CP_{1}P_{2}}+\sqrt{\eta_{2}^{2}}{|111\rangle }_{CP_{1}P_{2}}\right)⊗{|0\rangle }_{P_{3}}⊗\cdots ⊗{|0\rangle }_{P_{K}}. \end{array}$$

The entanglement gets counterfactually distributed between the server, $P_{1}$, and $P_{2}$. The success probability of the operation is $\eta_{1}^{2}/2$ in the absence of an AO and $\eta_{2}^{2}/2$ in its presence. The server repeats the same procedure with $P_{3}$ to $P_{N}$ by varying the switch *L*. If the photon of each participant comes out of their respective CQZ gate successfully, the final state of the system becomes

(8) $$\begin{array}{c} |\psi_{K}\rangle =\frac{1}{\sqrt{2}}\left(\sqrt{\eta_{1}^{K}}{|000...0\rangle }_{CP_{1}P_{2}...P_{N}}+\sqrt{\eta_{2}^{K}}{|111...1\rangle }_{CP_{1}P_{2}...P_{K}}\right) \end{array}$$

Finally, the K+1-partite GHZ state gets established between the server and the *K* participants.

**Figure 3:**
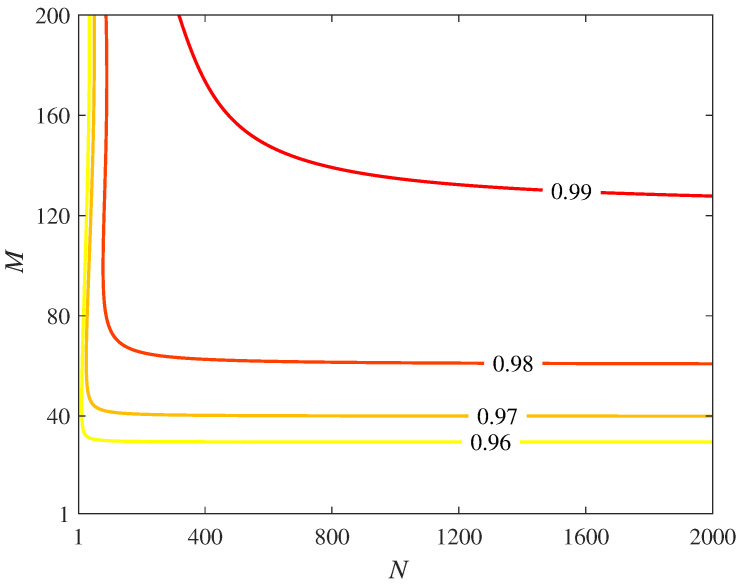
The success probability of counterfactual GHZ state distribution for a network of 50 participants where the CQZ gate held by each participant has N=2000 and M=200 of inner and outer cycles, respectively.

To achieve perfect GHZ state distribution, we can increase the number of inner and outer cycles of the CQZ gates. In Figure 3, we plot the success probability of counterfactual GHZ state distribution for 50 parties with 2000 inner and 200 outer cycles. It can be seen from the graph that as the values of *N* and *M* approach infinity, the values of $\eta_{1}^{K}$ and $\eta_{2}^{K}$ tend to 1, leading to perfect counterfactual GHZ state distribution.

### 2.3. Counterfactual Anonymous Quantum Teleportation (CAQT)

Most quantum communication protocols rely on the preshared entanglement to carry out the communication tasks. In the absence of preshared entanglement, communication between the individual parties cannot take place. In practice, preshared entanglement is severely degraded by the decoherence mechanism, resulting in mixed entangled states instead of pure entangled states. As a result, it has a detrimental influence on the performance of communication tasks [35]. In this article, we assume that each participant holds a single qubit, and no entanglement exists between them. Suppose that a network consists of a server and *K* participants where a quantum channel and classical authenticated channel exist between the server and each participant. By utilizing the method described in Section 2.2, all the participants in the network counterfactually create $J+\delta_{1}+\delta_{2}$ numbers of GHZ state among themselves.

**Figure 4:**
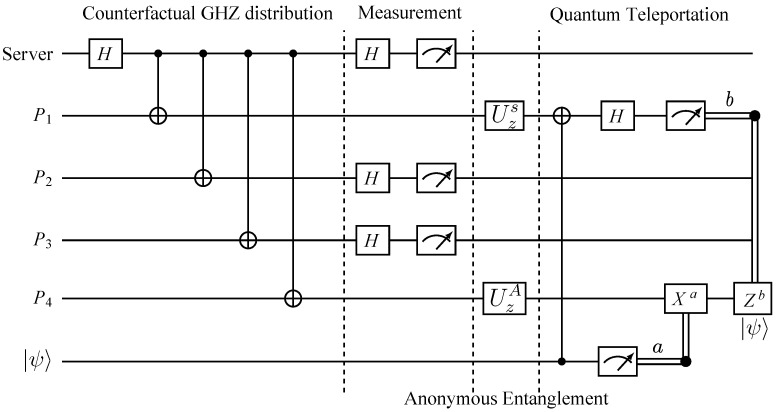
Circuit diagram of counterfactual anonymous quantum teleportation for a network consisting of a server and four participants $\left(P_{1},P_{2},P_{3}\;\mathrm{and}\;P_{4}\right)$. Here, we consider $P_{1}$ as the anonymous sender and $P_{4}$ as the anonymous receiver. $P_{1}$ anonymously wants to send the message |ψ〉 to their preferred receiver $P_{4}$ through the counterfactually distributed GHZ state. After the counterfactual GHZ state distribution, all participants except the sender and receiver measure their respective qubits in the X basis and announce the results through the classical channel while the sender and the receiver announce the random classical bit. Then, the sender performs $U_{z}^{s}$ on their qubit, where *s* denotes the random classical bit created by the server. On the other hand, the receiver performs $U_{z}^{A}$ on their qubit, where *A* represents the XOR value of the classical announcements of all the other participants except him. Once the anonymous entanglement is established between the sender and the receiver, they perform quantum teleportation in an anonymous manner based on that resource.

Anonymous quantum teleportation requires anonymous entanglement between the sender and the receiver. It requires an entangled channel between the sender and the receiver while their identities remain hidden from the rest of the network. In this setup, only one participant in the network can be the sender. Since multiple senders may be active simultaneously, they need to run the collision detection protocol described in Section 2.1.1 to avoid the failure of the protocol. When all the participants in the network get an agreement on the communication of one sender, the sender uses the notification protocol defined in Section 2.1.2 to inform the receiver anonymously. Afterward, the following steps are required to achieve the anonymous entanglement between the sender and receiver, as illustrated by the circuit diagram in Figure 4.

**Step 1:** For the anonymous entanglement to be reliable, it is necessary to check the security of the counterfactually distributed GHZ states. Different partite GHZ states collapse into other states when measured using a different basis. Thus, the server randomly chooses $\delta_{1}$ numbers of GHZ states and instructs the participants to measure their corresponding qubits using random basis $\left\{X,Z\right\}$ and announce their results. The server then determines whether or not the measurement results fall into the right form of K+1 GHZ basis. If the results are correct, the rest of the $J+\delta_{2}$ numbers of GHZ states are employed to establish the anonymous entanglement.

**Step 2:** For the remaining $J+\delta_{2}$ number of GHZ states, apart from the sender and receiver, every participant, including the server, measures their corresponding qubits in the X basis and stores the results as $\left\{m_{i}^{j}\right\}$ where *i* corresponds to the *i*th participant, *j* corresponds to the *j*th GHZ state and $j\in \left\{1,2,...,J\right\}$.

**Step 3:** The sender randomly creates a string $s=\left\{s^{1},s^{2},...,s^{J}\right\}$ and applies $U_{z}=|0\rangle \langle 0|-|1\rangle \langle 1|$ on their qubits if $s^{j}=1$.

**Step 4:** The receiver also randomly creates a string $r=\left\{r^{1},r^{2},...,r^{J}\right\}$.

**Step 5:** Using the anonymous broadcasting protocol described in Section 2.1.3, all the participants announce their classical message string through the broadcast channel. Then, the one who gets a notification, the receiver, calculates the XOR of all the broadcast classical message as follows:

(9) $$\begin{array}{c} A^{j}=r^{j}⊕\left(\overset{K+1}{\underset{i=11}{⨁}}m_{i}^{j}\right) \end{array}$$

If $A^{j}=1$, they perform $U_{z}$ on their qubit and the entanglement of the form $|\psi^{+}\rangle =|00\rangle +|11\rangle /\sqrt{2}$ has been anonymously distributed between the sender and receiver.

**Step 6:** To verify the created anonymous entanglement, for each round of anonymous entanglement creation, the sender randomly decides whether to use it for security checking or anonymous teleportation. In the case of security checking, the sender measures their qubit in X basis. Then, the server announces the measurement basis and sends the bit value 1 to the receiver via the classical broadcast channel, indicating that they are performing security checking. On the other hand, if the sender wishes to perform anonymous teleportation, they do not measure their qubit. Instead, they announce the random measurement basis and sends the bit 0 to the receiver via the classical broadcast channel. The receiver has to check the bit received from the classical broadcast channel before performing any measurement on their qubit. If it is 1, they measure their qubit using the announced basis. Otherwise, they do nothing on their qubit.

**Step 7:** Step 2 to 6 is run for $J+\delta_{2}$ times. If $\delta_{2}$ rounds of anonymous entanglement is chosen for security checking, the sender performs the quantum teleportation protocol on the remaining *J* numbers of anonymous entangled pairs.

## 3. Results and Discussion

### 3.1. Performance Analysis of CAQT in the Presence of Adversaries

In this section, firstly, we prove the correctness of our proposed protocol. Then, we analyze the security of the GHZ state that is counterfactually distributed among the participants, since it is the main step of our proposed protocol. Afterward, we prove the anonymity of the sender and receiver and the privacy of the teleported qubit in the presence of adversaries.

#### 3.1.1. Correctness of CAQT

Suppose that all the participants in the network are honest, and the perfect counterfactual entanglement gets established among them by using multiple numbers of *N* and *M* for each CQZ gate. Then, $\eta_{1}^{K}=\eta_{2}^{K}\approx 1$ and the state $|\psi_{K}\rangle$ becomes

(10) $$\begin{array}{c} |\psi_{K}\rangle \approx \frac{1}{\sqrt{2}}\left({|000...0\rangle }_{CP_{1}P_{2}...P_{K}}+{|111...1\rangle }_{CP_{1}P_{2}...P_{K}}\right). \end{array}$$

When all the participants in the network except the sender and receiver have performed X basis measurement on the shared counterfactual GHZ state in step 1, the state $|\psi_{K}\rangle$ transforms to

(11) $$\begin{array}{c} \begin{array}{cc} |\psi_{K}\rangle & \to I_{S}⊗I_{R}⊗X^{⊗K-2}\left(\frac{1}{\sqrt{2}}\left({|000...0\rangle }_{CP_{1}P_{2}...P_{K}}+{|111...1\rangle }_{CP_{1}P_{2}...P_{K}}\right)\right)\\ \; & =\frac{1}{\sqrt{2}}\left({|00\rangle }_{SR}+{\left(-1\right)}^{l}{|11\rangle }_{SR}\right)⊗\left(\overset{K+1}{\underset{i=1,i\neq S,R}{⨂}}|x\rangle \right), \end{array} \end{array}$$

where S (R) represents the sender (receiver), $l={⨁}_{i=1,i\neq S,R}^{K+1}\left|x\right|$, x∈{+,−} and +(−) denotes the value 0(1).

In the above Equation (11), if there is an odd number of |−〉 results in the X basis measurement, i.e., l=1, the entanglement shared between the sender and the receiver is ${|\phi^{-}\rangle }_{SR}=\frac{1}{\sqrt{2}}\left(|00\rangle -|11\rangle \right)$. Otherwise, they share the desired entanglement of the form ${|\phi^{+}\rangle }_{SR}=\frac{1}{\sqrt{2}}\left(|00\rangle +|11\rangle \right)$. Thus, the receiver must apply $U_{z}$ on their qubit if l=1 to get the desired shared entanglement. The result *A* calculated by the receiver in step 4 of our proposed protocol will agree with the value of *l*. The argument will be true regardless of the sender broadcasting 0 or 1. Hence, our protocol can provide the correct anonymous entanglement between the sender and the receiver.

#### 3.1.2. Security of Counterfactual GHZ State

The reliability of the anonymous entanglement in our proposed protocol depends on the counterfactual GHZ state. Thus, it is necessary to evaluate whether the adversaries become anonymously entangled with the valid K+1 counterfactual GHZ state. In the counterfactual context, the adversary (Eve) is accessible only to the quantum channel between the two end parties. Since only the H photon component, which carries no information, passes through the quantum channel in the H-CQZ gate, no information is available to Eve from the quantum channel [36]. Therefore, Eve cannot apply conventional attack strategies to get the quantum and classical side information. She must set up the counterfactual setting and attempt to perform possible attacks such as man-in-the-middle and Trojan horse attacks. We prove in the following that our proposed protocol is robust against the attacks introduced by Eve.

**Man-in-the-middle (MITM) attack:** This is the most common attack in most communication scenarios where Eve impersonates Alice for Bob and vice versa. To perform a MITM attack and mimic the setting of our proposed protocol, Eve pretends to be the server and prepares a QAO for the *K* participants. On the other hand, she also prepares *K* CQZ setups and acts as the *K* participants for the server. In case Eve can correctly guess the detection time window of the server and the path length between the server and the participants, the composite state after Eve’s attack becomes

(12) $$\begin{array}{c} \begin{array}{cc} {|\chi \rangle }_{mitm}= & \frac{1}{\sqrt{2}}\left(\sqrt{\eta_{1}^{K}}{|000...0\rangle }_{CE_{1}E_{2}...E_{K}}+\sqrt{\eta_{2}^{K}}{|111...1\rangle }_{CE_{1}E_{2}...E_{K}}\right)⊗\\ \; & \frac{1}{\sqrt{2}}\left(\sqrt{\eta_{1}^{K}}{|000...0\rangle }_{E_{S}P_{1}P_{2}...P_{K}}+\sqrt{\eta_{2}^{K}}{|111...1\rangle }_{E_{S}P_{1}P_{2}...P_{K}}\right), \end{array} \end{array}$$

where $E_{S}$ denotes Eve who pretends to be the server for the legitimate participants and $E_{k}$ denotes Eve who pretends to be the *k*th participant for the legitimate server where $k\in \left\{1,2,...,K\right\}$. From (12), we can see that the correlation between the actual server and the participants gets destroyed by Eve. The K+1 partite GHZ state may collapse into $2^{K}$ possible states when measured in the X basis. When the actual server and *K* participants measure their respective qubits in the presence of Eve in step 1 of our protocol, it results in two collapsed states. One originates from the K+1 partite GHZ state shared among the legitimate server *C* and $E_{k}$ participants, while the other originates from the K+1 partite GHZ state shared between $E_{S}$ and the legitimate *K* participants. The announced measurement result of the server comes from the former collapsed GHZ state while the results from the legitimate participants come from the latter collapsed GHZ state. This causes the measurement results of the server and the legitimate participants to fall into one of the $2^{K}+1$ possible result sets. However, the actual results must be one of $2^{K}$ possible result sets. On that account, the probability that the measurement results of the server and the legitimate participants fall into the valid GHZ basis is 1/2. Thus, we can indicate the probability of the existence of Eve in the channel by $\gamma_{mitm}=\frac{1}{2}.$

**Trojan horse (TH) attack:** To avoid being detected by the server, Eve builds a CQZ setup and exposes a ghost photon to the server to append her qubit to the GHZ state. A ghost photon here means that the chance of an eavesdropping photon appearing in the channel approaches zero due to the continuous measurement of the server during the CQZ operation [37]. This ghost photon can assist Eve in determining the presence or absence of an AO at the server from the detector click of their CQZ setting. However, a successful TH attack requires the eavesdropping photon to complete the operation within the access time window of the apparatus of the server. By varying the access time window, the server can discover the existence of Eve. If Eve is lucky enough, we can describe the resulting GHZ state after the attack as

(13) $$\begin{array}{c} {|\chi \rangle }_{th}=\frac{1}{\sqrt{2}}\left(\sqrt{\eta_{1}^{K+1}}{|000...00\rangle }_{CP_{1}P_{2}...P_{K}E}+\sqrt{\eta_{2}^{K+1}}{|111...11\rangle }_{CP_{1}P_{2}...P_{K}E}\right), \end{array}$$

where *E* denotes the qubit of Eve. We consider only one adversary in (13), which may change as the number of adversaries ($N_{E})$ increases. By tracing out Eve’s qubit, the density matrix of the legitimate system is

(14) $$\begin{array}{c} \begin{array}{cc} \rho_{GHZ}=Tr_{E}\left({|\chi \rangle }_{th}{\langle \chi |}_{th}\right)\; & =\sum_{i=1}^{K+1}p_{i}\rho_{C}⊗\rho_{P_{1}}⊗\rho_{P_{2}}⊗\cdots ⊗\rho_{P_{K}}\\ \; & \neq |\psi_{K}\rangle \langle \psi_{K}|, \end{array} \end{array}$$

where $p_{i}$ is the probability distribution. From (14), we can see that the tracing out of Eve’s system causes the legitimate system to collapse into a mixed state different from the expected shared GHZ state. In the presence of Eve, there are $2^{K+E}$ possible measurement results for the server and the *K* participants. Regardless of the number of Eve, only $2^{K+E-1}$ results will fall under the correct GHZ basis. Thus, when the server and the *K* participants perform security checking on the counterfactual GHZ state distribution in step 1, the probability that Eve cannot hide her presence is $\gamma_{th}=\frac{1}{2}$.

#### 3.1.3. Anonymity of CAQT

For anonymity, we consider two cases:

(i) Anonymity of the sender;
(ii) Anonymity of the receiver.

For the first case, we can identify the probability of a certain participant being the sender as in ref [7]

(15) $$\begin{array}{c} \mathrm{Prob}\left[S=s\right]=\frac{1}{n-t}, \end{array}$$

where *S* is the random variable identifying a sender and *t* is the number of corrupted participants. The global state between all participants is (8), with is symmetrically distributed between each participant. Similarly, the operations performed on the global state, i.e., measurements, are purely local. No party knows the operations performed by the other. The resultant state after a local operation is independent of the participant’s identity, which makes each participant equally likely to be the sender. Thus, the identity of the sender remains anonymous. We can adopt a similar reasoning for the second case.

An important observation is that any malicious participant can only alter the global state without identifying the identity of the sender and receiver. Similarly, even if the malicious participants collude, the malicious participants are unable to correctly identify the sender and receiver as long as the condition t≤K−2 gets fulfilled. This point is of great significance in our protocol as it shows that the resultant state created using counterfactual communication can guarantee anonymity.

#### 3.1.4. Privacy of Teleported Qubit

Although the main goal of anonymous communication is to protect the anonymity of the sender and receiver, it is also needed to assure the privacy of the transmitted quantum message. Once the security of the counterfactual GHZ state distribution is guaranteed, no external adversaries can get involved in the anonymous communication. Only two types of internal adversaries—semiactive adversaries and active adversaries—can lead to security flaws. We define a semiactive adversary as the one who follows the protocol but announces the wrong result rather than the correct one, and an active adversary as the one who does not follow the protocol and announces random results.

In the case of semiactive adversaries, an even number of adversaries still leads to the correct form of anonymous entanglement because their announcements of wrong measurement results cancel each other out and do not affect the anonymous entanglement phase. However, the server can detect an odd number of semiactive adversaries causing an invalid anonymous entanglement in step 6 of the protocol. Regardless of whether or not the correct anonymous entanglement gets established, the adversaries will not be able to extract the anonymous teleported quantum message. This limitation is due to the following two reasons: (i) the identity of the sender and the receiver is hidden from the adversaries, and (ii) only the notified receiver can correctly extract the two classical messages required for teleportation from the broadcast messages.

In the case of active adversaries, they attempt to entangle their qubits with the anonymous entanglement by not measuring their qubits in the X basis and broadcasting random results. If they are lucky enough and the random results are correct, they pass the security checking phase in step 6. Consequently, we can express the shared entanglement between the sender, receiver, and an active adversary as

(16) $$\begin{array}{c} |\Phi \rangle =\frac{1}{\sqrt{2}}{|000\rangle }_{srq}+{|111\rangle }_{srq}. \end{array}$$

where s, r, and q denotes the qubits of the sender, receiver, and active adversary, respectively. If ${|\psi \rangle }_{a}=\alpha {|0\rangle }_{a}+\beta {|1\rangle }_{a}$ is the quantum message the sender wants to teleport, the state after the Bell basis measurement of the sender becomes

(17) $$\begin{array}{c} \begin{array}{cc} {|\Phi \rangle }_{BM} & ={|\phi^{+}\rangle }_{as}\left(\alpha {|00\rangle }_{rq}+\beta {|11\rangle }_{rq}\right)+{|\phi^{-}\rangle }_{as}\left(\alpha {|00\rangle }_{rq}-\beta {|11\rangle }_{rq}\right)\\ \; & +{|\psi^{+}\rangle }_{as}\left(\alpha {|11\rangle }_{rq}+\beta {|00\rangle }_{rq}\right)+{|\psi^{-}\rangle }_{as}\left(\alpha {|11\rangle }_{rq}-\beta {|00\rangle }_{rq}\right) \end{array}. \end{array}$$

Still, the knowledge of adversaries is limited to the two classical messages required for teleportation. However, the receiver also cannot get the correct teleported qubit. Apart from disturbing the protocol, the adversaries cannot obtain any useful information from this attack. Hence, it is evident that the privacy of the teleported quantum message remains preserved in the presence of adversaries.

### 3.2. Performance Analysis of CAQT under Channel Noise

The most significant hurdle in any communication task is the presence of noise in the channel, which deteriorates the performance of communication. In this section, we investigate the impact of quantum noise in counterfactual anonymous quantum teleportation. We compare its performance with conventional anonymous quantum teleportation protocols achieved through GHZ states [7] and entanglement relays [8]. In conventional quantum communication, the qubit that passes through the quantum channel gets subjected to quantum-noise-induced alterations. Contrary to the conventional scenario, in counterfactual quantum communication, noise affects only the fraction of the qubit in path 2 that travels through the transmission channel, as shown in Figure 1.

The entanglement shared between the two end nodes lies at the heart of the quantum teleportation process. In our proposed protocol, the anonymous entanglement between the sender and receiver mainly relies on the counterfactual GHZ state distribution. Under the noisy quantum framework, the GHZ state shared among the participants is encompassed within the noise operator described by

(18) $$\begin{array}{c} \begin{array}{cc} \tilde{U}= & {{|1\rangle }_{C}{\langle 1|}_{C}}^{⊗K}⊗\left({⊗}_{i=1}^{K}I_{P_{i}}\right)⊗{I_{path}}^{⊗K}\\ \; & +{{|0\rangle }_{C}{\langle 0|}_{C}}^{⊗K}⊗\left({⊗}_{i=1}^{K}I_{P_{i}}\right)⊗{\left({|0\rangle }_{path}{\langle 0|}_{path}+{|1\rangle }_{path}{\langle 1|}_{path}\right)}^{⊗K}\\ \; & +{{|0\rangle }_{C}{\langle 0|}_{C}}^{⊗K}⊗\wedge_{P_{1}}⊗\wedge_{P_{2}}⊗\cdots ⊗\wedge_{P_{K}}⊗{{|2\rangle }_{path}{\langle 2|}_{path}}^{⊗K}, \end{array} \end{array}$$

where $I_{P_{i}}$ is a two-dimensional identity operator, $I_{path}$ is a three-dimensional identity operator, and $\wedge_{P_{i}}$ is the quantum noise operator encountered by the qubit of participant $P_{i}$ [36]. From the noise operator, we observe that the noise affects only the photon component that comes out of the CQZ gate and enters the transmission channel via path 2. The density operator of the noisy counterfactual GHZ state can be described as

(19) $$\begin{array}{c} \tilde{\rho_{GHZ}}=\tilde{U}{U_{CQZ}}^{⊗K}|\psi_{0}\rangle \langle \psi_{0}|{U_{CQZ}^{†}}^{⊗K}{\tilde{U}}^{†}, \end{array}$$

where $U_{CQZ}$ denotes the operation of the CQZ gate.

We can obtain the anonymous entangled state shared between *S* and *R* after step 4 by tracing out all participants in the network except *S* and *R* as follows:

(20) $$\begin{array}{c} \zeta_{SR}=\frac{1}{\kappa }Tr_{K-1}\left[\tilde{\rho_{GHZ}}\left(I_{SR}⊗|+\rangle {\langle +|}_{K-1}\right)\right] \end{array}$$

where κ is the normalization factor and $|+\rangle {\langle +|}_{K-1}$ is the projection onto the |+〉 state of K−1 participants. Note that, for the ideal noiseless channel, the anonymous entanglement shared between the *S* and *R* is $|\phi^{+}\rangle =\frac{1}{\sqrt{2}}\left(|00\rangle +|11\rangle \right)$. In the following sections, we compare the performance of the anonymous entanglement established using our CAQT protocol and conventional AQT protocols under different types of quantum noise. Here, we considered three types of quantum noise: dephasing noise, bit-flip noise, and depolarizing noise. To analyze the performance of noisy AQT protocols, the fidelity was employed as a metric to quantify the closeness between the ideal anonymous entanglement and the noisy anonymous entanglement.

#### 3.2.1. Comparison with Conventional AQT Protocols under Dephasing Noise

Dephasing is a process in which a qubit loses its phase information after traveling through a transmission channel. The action of the dephasing channel can be described as follows:

(21) $$\begin{array}{c} \rho \to \left(1-p\right)\rho +pZ\rho Z, \end{array}$$

where ρ is the density operator of the initial quantum state, *Z* is the Pauli Z operator, and 0≤p≤1 is a noise parameter.

**Figure 5:**
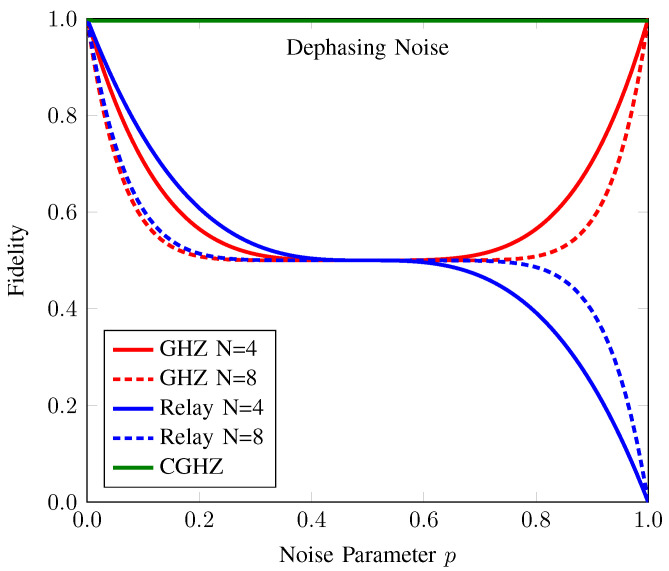
Fidelity of the anonymous entanglement of CAQT compared to those of conventional GHZ-state-based AQT and relay-based AQT under dephasing noise. Here, the dotted (solid) line denotes the fidelity of anonymous entanglement when the number of participants *N* is 4 (8). The fidelity values of the anonymous entanglement of conventional GHZ-state-based AQT (GHZ), relay-based AQT (Relay), and our counterfactual GHZ-state-based AQT (CGHZ) are represented by the red, blue, and green lines, respectively. The fidelity of our proposed protocol is the same for both N=4 and N=8.

In Figure 5, we compare our proposed protocol with the conventional GHZ-based and relay-based AQT protocols under the dephasing noise. In the conventional AQT protocols, the entanglement was preshared using ideal channel. For the consistency with our reasoning, we assumed that the entanglement was preshared among the participants under noise and there were N=K+1 participants in the network. For the GHZ-based AQT, the density operator of the initial resource can be written as $\tilde{\sigma_{GHZ}}=\wedge^{⊗N}|GHZ\rangle {\langle GHZ|}_{N}$. Without loss of generality, the anonymous entanglement $\Phi_{SR}$ arising from this preshared entanglement is described as

(22) $$\begin{array}{c} \Phi_{SR}=\frac{1}{N}Tr_{N-2}\left[\tilde{\sigma_{GHZ}}\left(I_{SR}⊗|+\rangle {\langle +|}_{N-2}\right)\right], \end{array}$$

where N is the normalization factor. The main difference between the conventional GHZ-based AQT and our proposed protocol is the distribution of the GHZ state. Since counterfactual communication is robust against dephasing noise [36], it allows a GHZ state distribution employing this property to remain unaffected by it as well. The fidelity of these protocols is plotted as a function of noise for N=4 and N=8 participants in Figure 5. We can see that the fidelity of the anonymous entanglement in our proposed protocol, $F_{AE}\left(\zeta_{SR}\right)=Tr\left[\zeta_{SR}|\phi^{+}\rangle {\langle \phi^{+}|}_{SR}\right]$, is almost equal to one regardless of the number of participants. On the other hand, for the conventional GHZ-based AQT, one can observe the parabolic behavior of the fidelity of the anonymous entanglement, $F_{AE}\left(\Phi_{SR}\right)=Tr\left[\Phi_{SR}|\phi^{+}\rangle {\langle \phi^{+}|}_{SR}\right]$.

In the relay-based AQT, each participant in the network holds a Bell pair. They perform entanglement swapping in a consecutive order to create entanglement between the first and last participants. Meanwhile, the sender and the receiver locally perform a CNOT operation on their target ancillary qubits before entanglement swapping with their next participant. Finally, a four-partite entanglement is formed between the sender, the receiver, and the first and last participants. When the first and last participants measure their qubit in the X basis, the anonymous entanglement is established between the sender and receiver. However, quantum noise affects each entanglement swapping between any two consecutive participants. Although this protocol enables long-distance communication, the quantum noise experienced in each entanglement swapping pair reduces the fidelity of the anonymous entanglement, $F_{AE}\left(\Psi_{SR}\right)=Tr\left[\Psi_{SR}|\phi^{+}\rangle {\langle \phi^{+}|}_{SR}\right]$. It is evident from the plot that in the presence of dephasing noise, $F_{AE}\left(\Psi_{SR}\right)$ decreases remarkably as the distance, i.e., the number of participants, increases under the noise parameter p≤0.5. Beyond that noise level, $F_{AE}\left(\Psi_{SR}\right)$ slightly increases as the number of participants increases. By contrast, our proposed protocol allows anonymous communication over remote participants with high fidelity, even in a large network with many participants. Hence, our proposed protocol outperforms the conventional AQT protocols in the presence of dephasing noise.

#### 3.2.2. Comparison with Conventional AQT Protocols under Bit-Flip Noise

Bit-flip noise flips the computational state of the qubit from |0〉 to |1〉 and vice versa. Given a bit-flip channel, it applies the identity operator with some probability 1−p and a bit-flip Pauli X operator with probability *p* on the incoming qubit. We can represent the generic model for the bit-flip channel as follows:

(23) $$\begin{array}{c} \rho \to \left(1-p\right)\rho +pX\rho X. \end{array}$$

**Figure 6:**
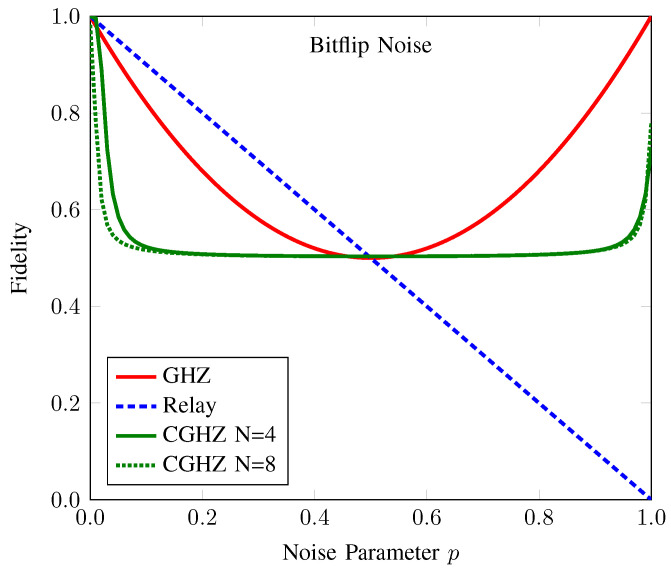
Fidelity of the anonymous entanglement of CAQT compared to those of conventional GHZ-based AQT and relay-based AQT under bit-flip noise. Here, the dotted (solid) line denotes the fidelity of anonymous entanglement when the number of participants *N* is 4 (8). The fidelity values of the anonymous entanglement of conventional GHZ-state-based AQT (GHZ), relay-based AQT (Relay), and our counterfactual GHZ-state-based AQT (CGHZ) are represented by the red, blue, and green lines, respectively. The conventional AQT protocols yield the same fidelity graph for both N=4 and N=8.

In Figure 6, we plot the fidelity values of the anonymous entanglement in our proposed protocol and the conventional AQT protocols under the bit-flip noise for N=4 and N=8. Within a noise range from 0 to 0.5, we can see that relay-based AQT performs better than the other two protocols. However, its performance degrades linearly with the noise beyond that range, whereas the GHZ-based AQT yields the best performance.

Although our proposed protocol is not the best option to choose in an environment with bit-flip noise, it can provide a fidelity greater than 0.5 for any noise level. It has been known that if the fidelity of an entanglement resource is greater than 0.5, it is considered a useful resource for quantum teleportation [9,38]. Hence, our proposed protocol is applicable for the anonymous transmission of a quantum message with the advantage of no information-carrying particle passing through the channel.

#### 3.2.3. Comparison with Conventional AQT Protocols under Depolarizing Noise

The depolarizing channel is the worst-case scenario among all the noise scenarios as it induces the combined effect of the dephasing and bit-flip channels. When the entangled state interacts with the environment under a depolarizing noise, it severely affects the entanglement feature of quantum states. Generally, it maps the pure input state to the mixed output state as follows:

(24) $$\begin{array}{c} \rho \to \left(1-p\right)\rho +p\pi , \end{array}$$

where π=I/2 is the maximally mixed state.

**Figure 7:**
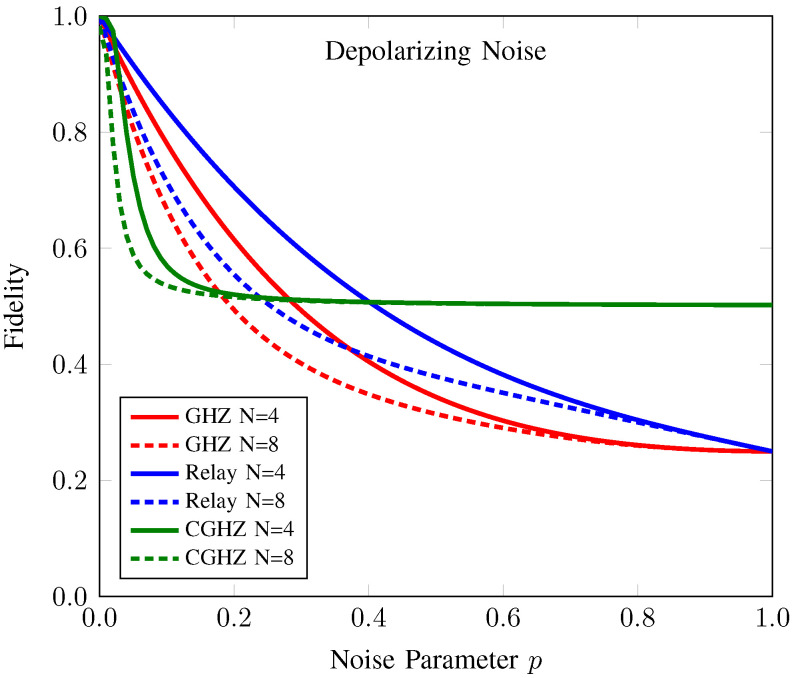
Fidelity of anonymous entanglement of CAQT compared to those of conventional GHZ-based AQT and relay-based AQT under depolarizing noise. Here, the dotted (solid) line denotes the fidelity of anonymous entanglement when the number of participants *N* is 4 (8). The fidelity values of the anonymous entanglement of conventional GHZ-state-based AQT (GHZ), relay-based AQT (Relay), and our counterfactual GHZ-state-based AQT (CGHZ) are represented by the red, blue, and green lines, respectively.

As shown in Figure 7, the fidelity of conventional protocols decreases as the number of participants and noise level increases. These protocols can support useful anonymous entanglement resources for teleportation only under a very low number of participants and noise levels. On the other hand, the fidelity $F_{AE}\left(\zeta_{SR}\right)$ of our proposed protocol reaches the saturation point at about 0.5 as the number of participants and noise level increases. Hence, in a large network with high noise levels, the performance of our proposed protocol surpasses that of conventional protocols and can provide useful resources for quantum teleportation.

To get a better inside on the behavior of the conventional AQT protocols and our proposed protocol, we provide a summary of comparison results in Table 1.

## 4. Application of CAQT in IoT Network

Secure IoT device communication is crucial for the reliable exchange of data in internet-enabled financial transactions, social communications, digitally signed documents, the transmission of medical data, or military communications [39,40]. Such applications require disparate network nodes performing computing, sensing, and data routing to collaborate and exchange huge quantities of data, which causes serious concerns for data security [39]. Attacks on privacy can reveal sensitive information such as the user identity and real-time user location data to malicious entities. The constraint on computational resources and power on individual nodes render postquantum cryptographic schemes ineffective [40].

Anonymous communication can offer information-theoretic anonymity for internode communication which is one of the requirements in IoT networks. Quantum-enabled solutions such as quantum anonymous transmission protocols can be fundamental to establishing security frameworks to support centralized and decentralized architectures for heterogeneous IoT applications in the long term. However, various environmental factors such as noise and loss due to faulty nodes, device reliability, and communication length may doom such protocols to be inefficient in real-world scenarios [41,42]. Our protocol, supported by the evidence provided above, outperformed the previously proposed protocols when channel noise and adversarial attacks were considered. Hence, it could support a plethora of application scenarios for single and multiple involved parties including anonymous wireless sensing networks, reliable social communication platforms, and telemedicine.

## 5. Conclusions

We presented an anonymous quantum teleportation protocol employing a counterfactual GHZ state distribution. We supplemented the protocol with a proof of its correctness and a comprehensive security analysis against potential attacks such as man-in-the-middle attacks and Trojan horse attacks, proving its robustness to malicious attacks. We demonstrated that it was simple to identify the presence of eavesdroppers in the quantum channel. Since the primary objective of anonymous communication is to protect the identities of the sender and the receiver, our proposed protocol met this criterion as long as the number of malicious participants was less than K−2. In addition, our protocol also preserved the privacy of the teleported qubit in the presence of adversaries. We further showed that our proposed protocol outperformed conventional GHZ-based AQT and relay-based AQT in the presence of dephasing noise and depolarizing noise. Although our proposed protocol did not offer the best performance under a bit-flip noise, it could provide useful quantum resources for anonymity. Thus, it is applicable in practical quantum communication scenarios.

## Figures and Tables

**Table 1:** Performance comparison of AQT protocols under three different types of channel noise.

Protocols		GHZ-Based AQT	Relay-Based AQT	Counterfactual GHZ-Based AQT
**Initial Resources**		**Preshared GHZ State**	**Bell States**	**Single Photons**
	Dephasing Noise	Parabolic curve	Nonlinear decrease	Not affected
Fidelity	Bit-flip noise	Parabolic curve	Linear decrease	High-order parabolic curve
	Depolarizing noise	High-order exponential decrease	Exponential decrease	Saturated at 0.5
	Dephasing Noise	Can provide	Cannot provide at p>0.5	Can provide
Useful quantum resource	Bit-flip noise	Can provide	Cannot provide at p>0.5	Can provide
	Depolarizing noise	Cannot provide at p>0.5	Cannot provide at p>0.5	Can provide

## Data Availability

Not applicable.

## Abbreviations

The following abbreviations are used in this manuscript:

GHZ	Greenberger–Horne–Zeilinger
QZ	Quantum Zeno
CQZ	Chained quantum Zeno
PBS	Polarizing beam splitter
SM	Switchable mirror
MR	Mirror
OD	Optical delay
OC	Optical circulator
D	Detector
AO	Absorptive object
QAO	Quantum absorptive object
AQT	Anonymous quantum teleportation
CAQT	Counterfactual anonymous quantum teleportation
